# Development and validation of a risk calculator for postoperative diplopia following orbital fracture repair in adults

**DOI:** 10.1038/s41598-024-54121-w

**Published:** 2024-02-13

**Authors:** Bashar Hassan, Nicholas Hricz, Seray Er, Joshua Yoon, Eric Resnick, Fan Liang, Robin Yang, Paul N. Manson, Michael P. Grant

**Affiliations:** 1 Division of Plastic and Reconstructive Surgery, R. Adams Cowley Shock Trauma Center, University of Maryland Medical Center, Baltimore, MD USA; 2 Department of Plastic and Reconstructive Surgery, Johns Hopkins Hospital, Baltimore, MD USA; 3 University of Maryland School of Medicine, Baltimore, MD USA; 4 Department of Surgery, George Washington University, Washington, DC USA; 5 Division of Plastic and Reconstructive Surgery, R. Adams Cowley Shock Trauma Center, University of Maryland Medical Center, 110 S Paca Street, Suite 4-S-124, Baltimore, MD 21201 USA

**Keywords:** Risk factors, Outcomes research

## Abstract

Postoperative diplopia is the most common complication following orbital fracture repair (OFR). Existing evidence on its risk factors is based on single-institution studies and small sample sizes. Our study is the first multi-center study to develop and validate a risk calculator for the prediction of postoperative diplopia following OFR. We reviewed trauma patients who underwent OFR at two high-volume trauma centers (2015–2019). Excluded were patients < 18 years old and those with postoperative follow-up < 2 weeks. Our primary outcome was incidence/persistence of postoperative diplopia at ≥ 2 weeks. A risk model for the prediction of postoperative diplopia was derived using a development dataset (70% of population) and validated using a validation dataset (remaining 30%). The C-statistic and Hosmer–Lemeshow tests were used to assess the risk model accuracy. A total of *n* = 254 adults were analyzed. The factors that predicted postoperative diplopia were: age at injury, preoperative enophthalmos, fracture size/displacement, surgical timing, globe/soft tissue repair, and medial wall involvement. Our predictive model had excellent discrimination (C-statistic = 80.4%), calibration (*P* = 0.2), and validation (C-statistic = 80%). Our model rules out postoperative diplopia with a 100% sensitivity and negative predictive value (NPV) for a probability < 8.9%. Our predictive model rules out postoperative diplopia with an 87.9% sensitivity and a 95.8% NPV for a probability < 13.4%. We designed the first validated risk calculator that can be used as a powerful screening tool to rule out postoperative diplopia following OFR in adults.

## Introduction

Orbital fractures represent up to 25% of traumatic facial injuries presenting to the emergency department^1^. There has been a rise in the frequency of orbital fractures over the years reaching up to 11.3 per 100,000 individuals in 2017^2,3^. Around 25% of orbital fractures often require surgical repair. The most commonly reported symptom following orbital fracture repair (OFR) is diplopia, which occurs in up to 52% of adults treated for orbital fractures^4,5^. Postoperative diplopia can be either residual i.e., persistent at 2 weeks or beyond following OFR, or incident diplopia i.e., newly experienced after to the resolution of eye swelling^5–7^. Due to the high occurrence of diplopia following OFR, it is essential for surgeons to be aware of the risk factors that might contribute to its occurrence and be able to predict the risk of this outcome preoperatively.

Currently, existing evidence on risk factors for diplopia following OFR is based on single-institution studies and small sample sizes^7–12^. Reported risk factors for postoperative diplopia include age older than 60 years^4,7,13^, extraocular muscle edema^9–11,14^, medial wall fractures^2,14–17^, fracture size^18,19^, and delayed surgery beyond 2 weeks of injury^7,18,20–22^. Furthermore, contributing factors to postoperative diplopia include missed diagnosis, ocular injury, and even an inadequate orbital wall reconstruction^23^. Although several risk factors for postoperative diplopia have been proposed, no risk calculator has been developed to help physicians predict this outcome.

Risk calculators utilize objective data to determine the risk of a certain outcome and promote informed decision making, proper patient management, and patient satisfaction^24–26^. A risk calculator for the prediction of diplopia following OFR would help physicians identify patients that would be at a higher risk for postoperative diplopia.

Herein, we conduct a retrospective cohort study to develop and validate the first risk calculator for the prediction of postoperative diplopia following OFR in adults. We hypothesize that severe fractures and delayed surgical repair are among the risk factors for postoperative diplopia which constitute a risk model that can predict this outcome with high accuracy. Our risk calculator will be of great benefit to surgeons and their patients as it will improve surgical planning and patient counseling.

## Methods

### Dataset

A retrospective chart review was performed at the R Adams Cowley Shock Trauma Center, University of Maryland Medical Center and the Johns Hopkins Hospital, Baltimore, Maryland from January 2015 to December 2019. This study adheres to the tenets of the Declaration of Helsinki. Inclusion criteria included patients who (1) were diagnosed with orbital fracture using computed topography (CT) scan, (2) underwent OFR at either institution, and (3) were followed up for at least 2 weeks after surgery. Excluded were patients who were under 18 years of age at the time of OFR, as well as patients whose follow-up period was less than 2 weeks postoperatively. Patient demographics, fracture and surgical characteristics, preoperative ocular symptoms, and postoperative outcomes were extracted and analyzed. The Institutional Review Board (IRB) of the University of Maryland and the Johns Hopkins University approved this study and waived informed consent regarding data collection.

### Outcomes and covariates

The primary outcome was the incidence and/or persistence of postoperative diplopia at least 2 weeks following OFR. Clinically significant postoperative diplopia was limited to at least 2 weeks following OFR, as transient and non-clinically significant postoperative diplopia is likely to resolve before then^5–7^. Preoperative diplopia at presentation and postoperative diplopia at follow-up were defined as double vision in any field of gaze which was clinically significant and affected daily function. Preoperative diplopia, postoperative diplopia, and ocular signs and symptoms were assessed through independent ophthalmology consultation at each visit.

The study sample was divided into patients with versus without postoperative diplopia. The two cohorts were compared based on: age at the time of injury, sex, race/ethnicity, alcohol use, medical comorbidities, mechanism of injury, surgical service, surgical timing, preoperative ocular symptoms, fracture severity, fracture site, and globe/soft tissue repair.

### Risk model development

Our risk model for the prediction of postoperative diplopia was derived using a random 70% sample of our study population. Bivariate analysis was performed to compare patients with versus without postoperative diplopia based on the aforementioned covariates. Multivariate logistic regression was performed to assess risk factors for postoperative diplopia accounting for covariates that had a *P*-value < 0.25 on bivariate analysis. The selection of the most parsimonious combination of risk factors predictive of postoperative diplopia was determined based on the *P*-value of variables and their interactions with other variables within the model. The reference group for preoperative ocular symptoms/signs e.g., enophthalmos and periorbital swelling, was the absence of these preoperative ocular symptoms/signs; fracture defect < 2 cm^2^ or displacement < 3 mm for fracture size/displacement; fracture repair < 2 weeks after injury for surgical timing; and no medial wall involvement for medial wall fracture. The strength of the association between the predictors and outcome was reported using adjusted odds ratio (aOR) and 95% confidence interval (CI). Statistical analysis was performed using IBM SPSS Statistics 28^27^. A *P*-value < 0.05 was considered significant.

### Risk model performance

The accuracy of our risk model was determined by testing its discrimination and calibration^28^ Discrimination is the model’s ability to distinguish between cases (with the outcome) and non-cases (without the outcome). This was assessed using a concordance statistic (C-statistic), also known as the area under the receiver operating characteristic (ROC) curve. The C-statistic values range from 0.5 (poor discrimination) to 1 (100% discrimination). In general, a larger C-statistic is associated with a more accurate model. Calibration refers to the model’s ability to correctly predict the outcome^29^. This was assessed using the Hosmer–Lemeshow test. Obtaining non-significance with this test indicates no significant difference between the observed vs predicted proportion of patients with postoperative diplopia, thus signifying good calibration^30^.

Two cut-off values for the probability of postoperative diplopia were determined. The first cut-off value is the probability under which postoperative diplopia is predicted with a 100% sensitivity i.e., none of the patients had postoperative diplopia. The second cut-off value was determined using Youden’s *J* index to maximize sensitivity and specificity, such that: *J* = *max* (sensitivity + specificity – 1)^31^. The sensitivity and specificity were determined at the Youden’s *J* index and the positive and negative predictive values were calculated.

Sensitivity is the probability of our model predicting diplopia in patients who actually had diplopia:

$$\frac{{Observed\,Diplopia}_{model\,prediction\,correct}}{({Observed\,Diplopia}_{model\,prediction\,correct}+{Absent\,Diplopia}_{model\,prediction\,incorrect})}$$

Specificity is the probability of our model predicting no diplopia in patients who indeed did not have diplopia:

$$\frac{{Absent\,Diplopia}_{model\,prediction\,correct}}{({Absent\,Diplopia}_{model\,prediction\,correct}+{Observed\,Diplopia}_{model\,prediction\,incorrect})}$$

Positive predictive value (PPV) represents the proportion of patients who actually had diplopia among those predicted to have diplopia by our model:

$$\frac{{Observed\,Diplopia}_{model\,prediction\,correct}}{({Observed\,Diplopia}_{model\,prediction\,correct}+{Observed\,Diplopia}_{model\,prediction\,incorrect})}$$

Negative predictive value (NPV) represents the proportion of patients who did not have diplopia among those predicted not to have diplopia by our model^32–34^:

$$\frac{{Absent\,Diplopia}_{model\,predicted\,correct}}{({Absent\,Diplopia}_{model\,predicted\,correct}+{Absent\,Diplopia}_{model\,predicted\,incorrect})}$$

### Risk model validation

Our risk model for the prediction of postoperative diplopia was derived from a random 70% sample of our study population (training dataset). Then, the model was validated by applying it to the remaining 30% of our study population (validation dataset) to estimate the probabilities of postoperative diplopia in that dataset. We also used the ROC curve to assess the accuracy of our risk model in predicting postoperative diplopia in the validation dataset. The model is considered validated in case the C-statistic, and thus the predictive accuracy, shows favorable results in both datasets. This validation method using similar C-statistic results has been previously described in the literature^35–38^.

### Risk model calculator

After risk model validation, the risk calculator was presented in the form of an interactive spreadsheet. The risk calculator accepts a numerical input regarding the patient’s age at the time of injury. All other inputs are binary for either the presence or absence of risk factors. The risk cut-off percent was determined using Youden’s *J* index, as previously discussed^31^. The risk calculator is based on the estimates of each of the risk factors in the model and the following standard binary logistic regression equation:

$$\mathrm{Estimated\,percent\,probability}=100\%*\frac{odds}{odds+1},$$

such that:

Odds = e^(age*0.02 − 0.87 if globe or other soft tissue repair was/will be performed—**1.25** if preoperative periorbital ecchymosis/swelling is present + **0.77** if preoperative enophthalmos is present + **0.69** if medial wall fracture is present + **1.27** if fracture is moderate-to-severe (> 2 cm^2^ defect or > 3 mm displacement) + **0.54** if orbital fracture repair was/will be performed > 2 weeks after injury − **3.15**).

Based on the above equation, Streamlit was used to develop an interactive, user-friendly online calculator (Streamlit, Version 1.28.0, 2024; available from https://www.streamlit.io/).

### Meeting presentation

This study’s abstract was presented as an oral presentation at the Plastic Surgery The Meeting (October 26–29, 2023) as a top scoring abstract and awarded the Outstanding Paper Presentation at the Craniomaxillofacial Abstract Session 11.

## Results

Of *n* = 254 patients included in our analysis, *n* = 51 (20.1%) had postoperative diplopia. Table 1 shows the distribution of patients according to the presence/absence of preoperative and postoperative diplopia. Of *n* = 100 patients who had preoperative diplopia, *n* = 26 (26%) had residual postoperative diplopia. Of *n* = 51 patients who had postoperative diplopia, *n* = 26 (51%) had preoperative diplopia (Table 1).

**Table 1:** Distribution of patients according to the presence/absence of postoperative and preoperative diplopia.

	Postoperative diplopia	No postoperative diplopia	Total of row cells
Preoperative diplopia	26	74	100
No preoperative diplopia	25	129	154
Total of column cells	51	203	254

### Bivariate analysis (70% dataset)

Table 2 shows the demographics, comorbidities, and fracture/surgical characteristics of our study population and compares them between patients who had postoperative diplopia vs patients who did not have postoperative diplopia within the 70% dataset. Of *n* = 183 patients included in the 70% dataset, postoperative diplopia was seen in *n* = 33 (18.0%) patients. Compared to patients who did not have postoperative diplopia, patients who did were significantly more likely to have had OFR after 2 weeks of injury (*n* = 43 [28.9%], *n* = 17 [51.5], *P* = 0.015), preoperative enophthalmos (*n* = 32 [21.3%], *n* = 15 [45.5], *P* = 0.004), moderate-to-severe fractures (*n* = 96 [64.0%], *n* = 29 [87.9%], *P* = 0.012), and less likely to have had preoperative periorbital ecchymosis/swelling (*n* = 130 [86.7%], *n* = 20 [60.6%], *P* < 0.001) (Table 2). Of *n* = 71 patients included in the 30% dataset, postoperative diplopia was seen in *n* = 18 (25.4%) patients.

**Table 2:** Patient demographics, comorbidities, orbital fracture and surgical characteristics of the study population, among patients who developed postoperative diplopia vs patients who did not using 70% of the study population.

	Overall (n = 254)	70% Dataset (n = 183)		*P*
		Postoperative diplopia (n = 33)	No postoperative diplopia (n = 150)	
Demographics				
Age, median (IQR), years	36.1 (27.8–50.7)	42.0 (31.3–50.2)	35.5 (27.5–50.9)	0.188
Male sex	184 (72.4)	24 (72.7)	111 (74.0)	1.000
Race and ethnicity				
White	114 (44.9)	16 (48.5)	66 (44.0)	0.878
Black	108 (42.5)	15 (45.5)	62 (41.3)	
Hispanic/Latino	11 (4.3)	1 (3.0)	7 (4.7)	
Asian	4 (1.6)	0 (0.0)	2 (1.3)	
Other	17 (6.7)	1 (3.0)	13 (8.7)	
Alcohol use	4 (1.6)	0 (0.0)	4 (2.7)	1.000
Comorbidities				
Hypertension	65 (25.6)	11 (33.3)	40 (26.7)	0.520
Diabetes mellitus	19 (7.5)	2 (6.1)	12 (8.0)	0.750
Eye disorders e.g., cataract, visual symptoms, glaucoma	7 (2.8)	1 (3.0)	4 (2.7)	1.000
Orbital fracture and surgical characteristics				
Mechanism of injury				
Fall	52 (20.5)	5 (15.2)	31 (20.7)	0.311
Assault	102 (40.2)	18 (54.5)	57 (38.0)	
Sports-related	20 (7.9)	1 (3.0)	12 (8.0)	
Motor vehicle collision	46 (18.1)	4 (12.1)	30 (20.0)	
Pedestrian struck	4 (1.6)	0 (0.0)	3 (2.0)	
Bicyclist struck	1 (0.4)	0 (0.0)	1 (0.7)	
Gun	10 (3.9)	0 (0.0)	7 (4.7)	
Motorcycle collision	1 (0.4)	0 (0.0)	1 (0.7)	
Unknown/other	18 (7.1)	5 (15.2)	8 (5.3)	
Surgical service				
Plastic surgery	120 (47.2)	17 (51.5)	73 (48.7)	0.974
ENT	23 (9.1)	2 (6.1)	14 (9.3)	
OMFS	21 (8.3)	2 (6.1)	10 (6.7)	
Oculoplastics	90 (35.4)	12 (36.4)	53 (35.3)	
Isolated orbital fracture	86 (33.9)	13 (39.4)	50 (33.3)	0.547
Two fractured orbits	23 (9.1)	2 (6.1)	13 (8.7)	1.000
Surgical timing				
Within 2 weeks of injury	166 (65.6)	16 (48.5)	106 (71.1)	**0.015**
> 2 weeks of injury	87 (34.4)	17 (51.5)	43 (28.9)	
Preoperative ocular symptoms				
Extraocular muscle entrapment	13 (5.1)	1 (3.0)	7 (4.7)	1.000
Limited ocular motility	70 (27.6)	12 (36.4)	42 (28.0)	0.400
Enophthalmos	66 (26)	15 (45.5)	32 (21.3)	**0.004**
Diplopia	100 (39.4)	14 (42.4)	53 (35.3)	0.550
Periorbital ecchymosis/swelling	204 (80.3)	20 (60.6)	130 (86.7)	** < 0.001**
Orbital fracture severity				
Mild (< 2 cm^2^ defect or < 3 mm displacement)	82 (32.3)	4 (12.1)	54 (36.0)	**0.012**
Moderate-to-severe (> 2 cm^2^ defect or > 3 mm displacement)	97 (38.2)	29 (87.9)	96 (64.0)	
Orbital fracture site				
Roof	24 (9.4)	1 (3.0)	14 (9.3)	0.314
Lateral wall	40 (15.7)	5 (15.2)	25 (16.7)	1.000
Floor	230 (90.6)	31 (93.9)	133 (88.7)	0.534
Medial wall	123 (48.4)	21 (63.6)	71 (47.3)	0.123
Globe or soft tissue repair	45 (17.7)	3 (9.1)	29 (19.3)	0.209

Frequency data are reported as No. (%).

Percentages shown were calculated as a fraction of respective groups (*n* = 254: all patients; *n* = 33: patients who developed postoperative diplopia within the 70% dataset; *n* = 150: patients who did not develop postoperative diplopia within the 70% dataset).

Significant *P*-values < 0.05 are bolded.

*ENT* ear, nose, and throat; *OMFS* oral and maxillofacial surgery; *IQR* interquartile range.

### Multivariate analysis (70% dataset)

Table 3 shows the multivariate logistic regression analysis which yielded the risk model for the prediction of postoperative diplopia. The risk model consisted of the following demographics and surgical characteristics: age at injury, globe or soft tissue repair, periorbital ecchymosis/swelling, medial wall fracture, preoperative enophthalmos, orbital fracture severity, and surgical timing. Moderate-to-severe fractures (> 2 cm^2^ defect or > 3 mm displacement) were significantly associated with greater odds of postoperative diplopia (aOR [95% CI] 3.548 [1.106–11.381]) (Table 3).

**Table 3:** Estimates, standard errors, and variables associated with postoperative diplopia in multivariate logistic regression analysis using the 70% dataset.

	Estimate	SE	aOR	95% CI
Intercept	− 3.15	0.98	–	–
Age	0.02	0.01	1.02	0.99–1.05
Globe/other soft tissue repair	− 0.87	0.67	0.42	0.11–1.56
Preoperative periorbital ecchymosis/swelling	− 1.25	0.47	0.29	**0.11–0.72**
Medial wall fracture	0.69	0.46	2.00	0.82–4.87
Preoperative enophthalmos	0.77	0.47	2.16	0.86–5.42
Fracture defect > 2 cm^2^ or displacement > 3 mm	1.27	0.60	3.55	**1.11–11.38**
Orbital fracture repair > 2 weeks after injury	0.54	0.47	1.71	0.68–4.28
C-statistic	0.804			
Hosmer–Lemeshow	0.199			

Significant 95% confidence intervals are bolded.

Reference groups were as follows: no globe or other soft tissue repair, no preoperative periorbital ecchymosis/swelling, no medial wall fracture, no preoperative enophthalmos, mildly severe fractures (< 2 cm^2^ defect or < 3 mm displacement), orbital fracture repair < 2 weeks after injury.

*SE* standard error, *aOR* adjusted odds ratio, *CI* confidence interval, *C-statistic* concordance statistic or the area under the receiver operating curve.

### Development and performance of risk model

The risk model in Table 3 was derived using the 70% dataset and contained all covariates with a *P* < 0.25 on bivariate analysis.

**Figure 1:**
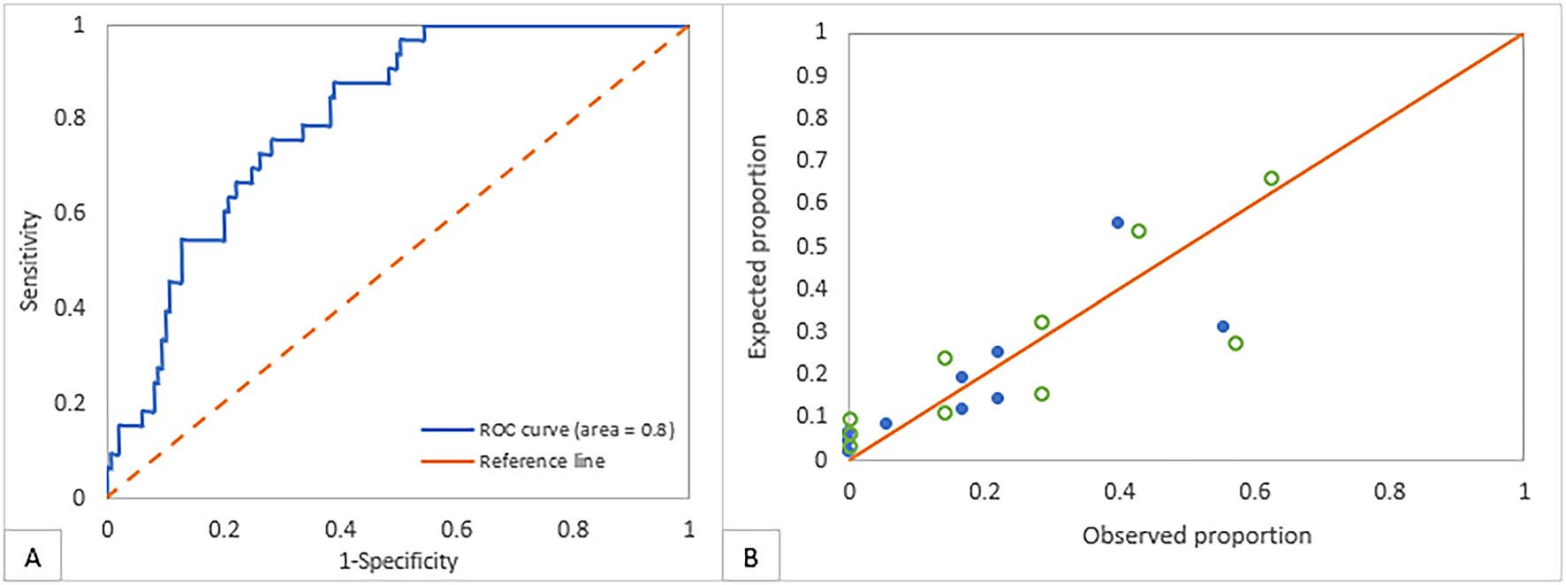
(**A**) The receiver operating characteristic (ROC) curve of the predictive model with an area under the curve = 80.4% (suggesting very good discrimination), sensitivity = 87.9%, and specificity = 61.1%. (**B**) The calibration curve of the predictive model showing the degree of consistency between the observed proportion and the expected proportion of patients with postoperative diplopia using the predictive model in both the development and validation datasets (Full circles indicate datapoints from the development dataset; Empty circles indicate datapoints from the validation dataset; Hosmer–Lemeshow test *P* > 0.05 suggesting goodness-of-fit).

Figure 1A shows the receiver operating characteristic (ROC) curve of the risk model. The C-statistic was 0.804 using the 70% dataset indicating very good discrimination. Figure 1B shows the calibration curve of the risk model. The figure demonstrates the high degree of consistency between the observed proportion and the expected proportion of patients with postoperative diplopia using the risk model in both the development and validation datasets (Hosmer–Lemeshow test *P* > 0.05 suggesting goodness-of-fit).

The probability cut-off value under which none of the patients had postoperative diplopia was 8.9%. The *J*-point was 0.49 and the corresponding optimal risk cut-off percent was 13.4%. The corresponding sensitivity and specificity were 87.9% and 61.1%, respectively. The PPV and NPV were 33.3% and 95.8%, respectively. Table 4 shows the frequency of both predicted and observed postoperative diplopia and demonstrates how the PPV and NPV were derived. Both the sensitivity and NPV of our risk model were excellent, signifying its usefulness as a screening tool to rule out postoperative diplopia.

**Table 4:** Distribution of patients according to the observed and predicted postoperative diplopia using the 70% dataset.

	Observed postoperative diplopia		Total of row cells
	Present	Absent	
Predicted postoperative diplopia			
Present	29	58	87
Absent	4	92	96
Total of column cells	33	150	183

Sensitivity = 29/33 = 87.9%, correlates with Negative Predictive Value = 92/96 = 95.8%

Specificity = 92/150 = 61.1%, correlates with Positive Predictive Value = 58/87 = 33.3%

### Risk model validation

The risk model was then applied to the 30% validation dataset. The C-statistic of the risk model used to estimate postoperative diplopia in the 30% dataset was 0.79, indicating very good discrimination. This result indicates that the risk model maintained its discriminatory power in an independent dataset, and that the risk model’s performance was very similar in both datasets.

### Development of risk calculator

The risk model can be used as an interactive risk calculator, as described in the methods. Values can be entered as 0 for absence, and 1 for presence of a specific variable. The only continuous variable in the risk model was age, whose values can be entered as a number. The generated value of the risk calculator represents the risk percentage of postoperative diplopia at least 2 weeks after OFR.

The risk calculator is available online and can be accessed via the following link: https://riskcalculatorforpostoperativediplopia.streamlit.app/

Below we present some hypothetical scenarios of patients planned for OFR to determine the risk percentage of postoperative diplopia:

1. Thirty-five-year-old patient, presenting after trauma without enophthalmos or periorbital swelling, fracture defect < 2 cm^2^ and displacement < 3 mm with no medial wall involvement. OFR planned to be performed in > 2 weeks without globe or soft tissue repair: **12.85%.** According to our risk model, there is a > 96% probability that this patient will not have postoperative diplopia (< 13.4%).
2. Same as case #1 but with fracture defect > 2 cm^2^ and displacement > 3 mm: **34.34%.** This signifies the impact of fracture severity on increasing the risk of postoperative diplopia.
3. Same as case #2 but with OFR planned to be performed in < 2 weeks of injury: **23.43%.** This signifies the impact of early surgical repair on decreasing the risk of postoperative diplopia.
4. Twenty-five-year old patient, presenting after trauma without preoperative enophthalmos or periorbital swelling, fracture < 2 cm^2^ and < 3 mm in displacement without medial wall involvement. OFR planned to be performed in < 2 weeks without globe repair: **6.54%.** According to our risk model, there is a 100% probability that this patient will not have postoperative diplopia (< 8.9%).

## Discussion

The purpose of this multi-center study was to design a risk calculator to determine the probability of postoperative diplopia after OFR. We utilized 70% of our study population to derive the risk model which was validated against the remaining 30%. Our risk model was designed to capture both residual diplopia persisting beyond two weeks, as well as new diplopia experienced after swelling resolution within the same period. This risk calculator will better prepare physicians by allowing them to identify and optimize the management of high-risk individuals presenting with orbital fractures.

Our risk model was made up of seven risk factors associated with postoperative diplopia: age at injury, preoperative enophthalmos, preoperative periorbital swelling, fracture severity based on defect size and fracture displacement, surgical timing, repair of globe/soft tissue, and medial wall involvement. When considered together, only fracture severity (defects > 2 cm^2^ or displacement > 3 mm) was a significant risk factor for postoperative diplopia. Age was not significantly associated with postoperative diplopia, which was consistent with the findings of Leitch et al.^4^. However, this contrasts with the results of Hosal et al. who found a significant association between older age and postoperative diplopia^7^.

The presence of preoperative enophthalmos was the only preoperative symptom that was associated, albeit not significantly, with increased odds of postoperative diplopia. Jin et al. also reported a nonsignificant association between preoperative enophthalmos and postoperative diplopia in their retrospective cohort study of 63 patients^10^.

Delayed OFR > 2 weeks after injury was not significantly associated with greater odds of postoperative diplopia compared to patients who had surgery before. This contrasts with prior literature showing that earlier OFR, within 8 days of injury, is associated with greater odds of postoperative diplopia compared to OFR performed later^21^. Dal Canto and Linberg found no significant difference in ocular motility, diplopia, and time to resolution of diplopia between patients treated within 2 weeks of injury compared to those treated within 2 to 4 weeks of injury^38^.

Medial wall involvement was also not significantly associated with greater odds of postoperative diplopia. Biesman et al. found that medial wall involvement was associated with greater odds of postoperative diplopia, compared to isolated floor fractures, probably to the greater degree of difficulty in restoring the preoperative contour of orbits with combined fractures^2^.

The strongest and only significant predictor for postoperative diplopia was fracture severity. Patients with fracture defects > 2 cm^2^ or displacement > 3 mm had nearly four times the odds of postoperative diplopia compared to those who had less severe fractures. There is a paucity of literature studying the association between fracture severity and postoperative outcomes following OFR. Hawes et al. showed that patients with large fractures (≥ 15 fracture volume units or > one-half floor fractured) were significantly more likely to develop postoperative extraocular muscle dysfunction and enophthalmos compared to patients with smaller fractures^18^. No studies have found an association between fracture severity and postoperative diplopia.

On the other hand, our predictive model showed that preoperative periorbital swelling was significantly associated with lower odds of postoperative diplopia. The rationale for this is that preoperative periorbital swelling may be significant enough to occlude vision in one eye or hinder double vision temporarily. No significant association was found between periorbital swelling and postoperative diplopia by Jin et al.^10^. The repair of the globe or other soft tissue repair was also associated, albeit not significantly, with lower odds of diplopia in our study population. Similarly, repair of the globe or soft tissue repair was likely a protective factor against diplopia due to patients likely being monocular and unable to report double vision.

There is a hypothesis that the specific type of implants used, such as porous polyethylene or titanium, might affect the risk of postoperative diplopia^39^. A comparative study between two commonly used implants, the DePuy/Synthes titanium MatrixMIDFACE prefabricated implants and the porous polyethylene/titanium hybrid implants, conducted by one of our senior authors, found no notable difference in the incidence of postoperative diplopia^40^. Therefore, the type of reconstructive material was not included as a variable in the development of our risk calculator.

Our risk model demonstrated excellent sensitivity and NPV of 87.9% and 95.8%, respectively, for a cut-off value of 13.4%. Hence, for any predicted probability of postoperative diplopia less than 13.4%, we are more than 96% confident that the patient will not have diplopia 2 weeks after OFR. In particular, for any predicted probability of postoperative diplopia less than 8.9%, we are 100% confident that the patient will not have diplopia 2 weeks after OFR. As the predicted probability increases more than 13.4%, the risk of postoperative diplopia increases, but the validity of the risk calculator decreases. This is due to the relatively lower specificity and PPV: 61.1% and 33.3%, respectively. This would mean that for any predicted probability of postoperative diplopia greater than 13.4%, we are less than 96% confident that the patient will actually develop diplopia. This highlights the usefulness of our risk calculator as a screening tool to predict the *absence* of and rule *out* postoperative diplopia, rather than confirming the presence of and ruling in postoperative diplopia 2 weeks after OFR. Additionally, the interactive online calculator we built is user-friendly and enable clinicians to efficiently calculate the risk of postoperative diplopia.

If used preoperatively, our risk calculator can guide proper management and surgical planning of patients presenting with orbital fractures to minimize the risk of postoperative diplopia. For example, according to our risk calculator, a 25-year-old patient with preoperative enophthalmos, no perioperative swelling or medial wall involvement, fracture size < 2 cm^2^ or displacement < 3 mm, and OFR planned to occur > 2 weeks after injury would have a 20.5% risk of postoperative diplopia. However, the same patient would have a 13.1% risk of postoperative diplopia if OFR were to occur < 2 weeks after injury. Hence, our risk calculator can help surgeons in surgical planning and management of patients presenting with orbital fractures. Our risk calculator can also be used postoperatively to counsel patients and communicate realistic expectations.

Our study is not without limitations. First, our risk calculator is excellent for ruling out postoperative diplopia (predicted probability less than 13.4%) but is less accurate as the predicted probability gradually rises above 13.4%. Hence, it is more accurate in ruling out than ruling in postoperative diplopia. Second, our study is a retrospective cohort which limits our analysis to available data in medical charts. Manual data extraction is prone to error. However, this was mitigated by having two independent authors for data collection and a third author for conflict resolution. Third, we limited our follow-up period to at least 2 weeks following OFR to capture clinically significant postoperative diplopia. However, we relied on previously published data to choose this cut-off value^5–7^. The multi-center nature of our study provides greater generalizability compared to single institution studies, but generalizability remains limited given that both centers are located within the same city. Nonetheless, the large sample size of our study provides our results with great power. Larger datasets are needed to further validate our risk calculator in the future.

Hence, we designed the first validated risk calculator that can be used as a powerful screening tool to rule out postoperative diplopia following OFR in adults. This will improve surgical planning and management of patients presenting with orbital fractures.

## Data Availability

The dataset generated during and/or analysed during the current study is not publicly available due to identifying information within the dataset. Furthermore, having our dataset publicly available was not part of our IRB acceptance. However, the dataset may be available from the corresponding author on reasonable request.
